# Design of a Novel Class of *N*-Heterocyclic Carbene Cycloplatinated Complexes Containing Pyrene Chromophores

**DOI:** 10.3390/molecules30224473

**Published:** 2025-11-19

**Authors:** Zeping Zhang, Yaping Cheng, Geoffrey Gontard, Tim Riesebeck, Sandy Fornal, Thomas Strassner, Hani Amouri

**Affiliations:** 1 Institut Parisien de Chimie Moléculaire (IPCM), UMR CNRS 8232, Sorbonne Université-Campus Pierre et Marie Curie, 4 Place Jussieu, CEDEX 05, 75252 Paris, France; 2 Physikalische Organische Chemie, Technische Universität Dresden, 01069 Dresden, Germany

**Keywords:** luminescent platinum complexes, *N*-heterocyclic carbenes, red emitters, X-ray structural determination

## Abstract

Cycloplatinated complexes incorporating pyrene chromophores of the formulae (C^C*)Pt(acac) (**3**, **4**), (C^C* = Pyrenyl-NHC, acac = acetylacetonate) were prepared and fully characterized. For comparison, two regioisomeric complexes were prepared following synthetic procedures developed by us. One isomer has the Pt(II) center attached to the 2-position of the pyrene chromophore, while the other regioisomer has the metal center attached at the 1-position of the organic chromophore. The molecular structures of **3** and **4** were ascertained by X-ray diffraction, and they prove the identity of the targeted compounds. Both complexes are emissive at room temperature in the red part of the spectrum in poly(methyl methacrylate) (PMMA), as well as at 77 K in 2-methyltetrahydrofuran (2-MeTHF). The regioisomer containing the Pt(II) at the 1-position shows enhanced emissive properties compared to the other regioisomer.

## 1. Introduction

Square planar cyclometalated platinum complexes, containing chelating (C^N) aromatic ligands (e.g., 2-arylpyridine), have been the focus of intense investigations in recent years [1,2]. The strong donor effect of the aromatic carbon atom, along with the chelation, generates stable phosphorescent complexes. Replacement of the (C^N) ligands by (C^C*) chelating ligands increases the energy barrier between ^3^MLCT (metal-to-ligand charge transfer) and ^3^MC (metal-centered) states, suppressing nonradiative decay and thus improving the phosphorescence efficiency [1,3]. This can be achieved by introducing *N*-heterocyclic carbenes (NHCs) within the ligand framework [4,5,6]. Square planar platinum complexes containing the (C^C*) aromatic–NHC ligand have been well investigated by some of us, and we demonstrated that such compounds can be used to engineer blue OLEDs with high quantum yields [7,8,9,10,11,12].

Despite this success, less is known about related platinum complexes containing (C^C*) pyrene–NHC ligands. However, we note that some square planar platinum complexes containing pyrene chromophores, but without NHCs ligands, have been described [13,14].

*N*-heterocyclic carbenes (NHCs) are well known for their strong σ-donating properties, which make them interesting ligands for various applications in almost all areas of chemistry, such as organometallics, coordination chemistry, catalysis and medicinal chemistry [15,16,17,18,19]. More recently, efforts have been devoted to the use of such ligands to design stable luminescent organometallic and coordination complexes [1,6,20]. This is driven by the fact that NHC ligands tend to push the ^3^dd dark states to a higher energy, and hence avoid the deactivation processes of the low-lying MC (metal-centered) transition states. In a previous study, we prepared some halogenated coinage metal complexes featuring pyrene-NHC chromophoric ligands [21,22], which turned out to be blue emitters [23]. The use of an organic chromophore enhances the emissive properties of these compounds, which otherwise are weakly or even non-emissive complexes [24].

Pursuing our work in this field, we envisioned preparing a new class of luminescent carbene complexes incorporating Pt(acac) as an inorganic chromophore and pyrene as an organic chromophore. Pyrene has a low-lying triplet state that is not accessible at room temperature in the free ligand [25,26,27,28,29]. The platinum center, with its high spin-orbit coupling constant, enables access to the triplet manifold of the organic chromophore at room temperature, generating red to near-infrared (NIR) luminescent complexes. In previous work, we successfully employed this approach to obtain red and NIR complexes using cyclometalated iridium complexes and naphthalimide chromophores [30,31,32]. NIR emissive materials have received considerable attention due to their applications in the biological domain and in dynamic therapy [33,34].

**Figure 1:**
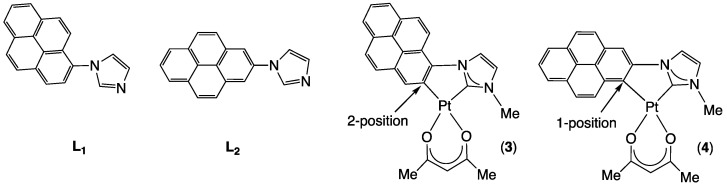
Imidazole pyrenyl ligands (**L_1_** and **L_2_**). Novel (C^C*)Pt(acac) (**3**, **4**), regioisomeric complexes described in this work. (C^C* = Pyrenyl-NHC).

Here, we report on the synthesis of some luminescent NHC-Pt(II) complexes containing a pyrene luminophore. The regioisomeric effect of the Pt(II) substitution at the 1- or 2-position of the pyrene chromophore was investigated for their impact on the emissive properties of these novel classes of compounds (Figure 1).

## 2. Results and Discussion

### 2.1. Synthesis and Characterization

**Scheme 1:**
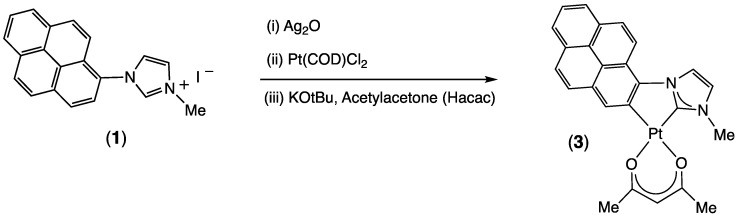
Synthesis of pyrenyl–NHC–Pt(II)(acac) complex (**3**).

The preparation of the imidazole–pyrenyl ligand **L_1_** and its related azolium salt **1** was performed according to a synthetic procedure reported previously by our group [22,23]. The synthesis of NHC–pyrenyl Pt(II) complex **3** was performed according to the one-pot synthesis procedure developed by Strassner et al. [7,35,36,37,38]. Thus, *N*-Me-pyrenyl azolium salt **1** was reacted with silver(I) oxide in dry dimethylformamide (DMF) to provide the silver(I)–carbene complex in situ, presumably followed by transmetalation to the dichloro(1,5-cyclooctadiene)platinum(II) [Pt(COD)Cl_2_] metal precursor. Cyclometalation occurred at elevated temperatures, and treatment with the acetylacetonate (acac) auxiliary ligand under basic conditions using potassium *tert*-butanolate (KOtBu) as the base (Scheme 1) provided target compound **3** in a 21% yield.

The ^1^H-NMR of the pyrenyl–NHC–Pt(acac) complex (**3**) recorded in dichloromethane (CD_2_Cl_2_) demonstrated the disappearance of the characteristic acidic NC*H*N proton of the azolium salt (**1**) at around δ 10 ppm, confirming the formation the NHC–Pt bond. The pyrene aromatic protons appear as several multiplets in the range of δ 7.7–8.9 ppm due to the lack of symmetry after the cycloplatination process and the appearance of satellite peaks due to the coupling with ^195^Pt center. The ^13^C-NMR of the carbene carbon atom for **3** displays a signal at around δ 185 ppm after prolonged acquisition.

In order to obtain regioisomeric complex **4** we first prepared novel imidazole-pyrene ligand **L_2_** from imidazole and 2-bromo-pyrene. The latter is not commercially available and was prepared following a modified synthetic procedure [39]. The carbene-precursor azolium salt (**2**) was obtained via the treatment of **L_2_** with MeI and isolated as off-white salt in a 92% yield.

**Scheme 2:**
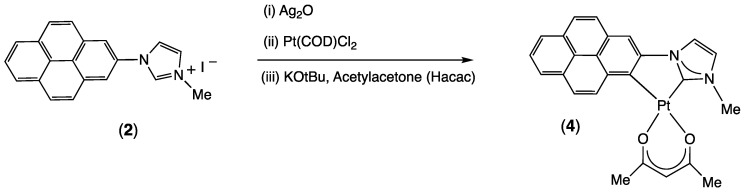
Synthesis of pyrenyl–NHC–Pt(II)(acac) complex (**4**).

The pyrenyl–NHC–Pt(acac) complex (**4**) was prepared in an analogous way to the preparation of complex **3** and after purification was obtained as an off-white solid in a 19% yield (Scheme 2).

The identity of the starting materials and of target complexes **3** and **4** was additionally confirmed by electrospray mass spectrometry. Full characterizations (^1^H, ^13^C, MS) are given in the experimental section and in the Supplementary Materials (Figures S1–S8). Moreover, the molecular structures of **3** and **4** were ascertained by a single-crystal X-ray diffraction study (vide infra).

### 2.2. X-Ray Molecular Structures of (Pyrenyl-NHC-Me)-Pt(acac) Isomers 3 and 4

**Figure 2:**
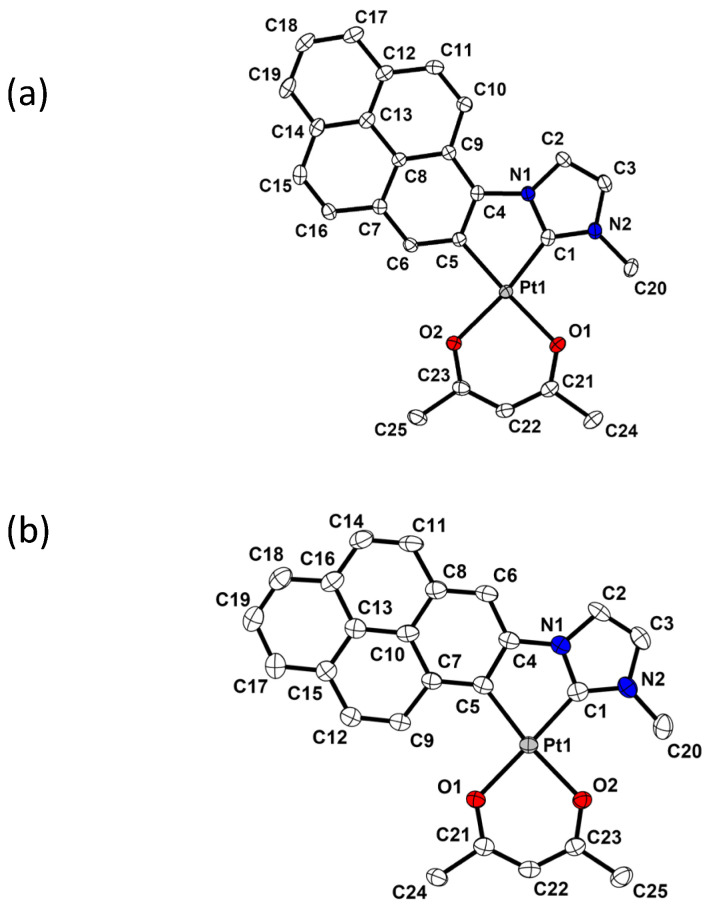
Crystal structures of **3** (**a**) and **4** (**b**). Thermal ellipsoids were drawn at 30% probability. Hydrogens were omitted for clarity. Selected bond distances (Å) and angles (°). For complex **3**, Pt1-C1 = 1.937(3), Pt1-C5 = 1.981(3), Pt1-O1 = 2.087(2), Pt1-O2= 2.066(2), O1-C21= 1.263(4), O2-C23 = 1.279(4), C1-Pt1-C5 = 79.33(12), C1-Pt1-O1 = 172.22(10), C1-Pt1-O2 = 172.22(10), C5-Pt1-O2 = 93.04(10), O1-Pt1-O2 = 89.59(8), C1-N1-C2/C4-C5-C6 = 10.9(3). For complex **4**: Pt1-C1 = 1.956(6), Pt1-C5 = 2.034(6), Pt1-O1 = 2.057(4), Pt1-O2= 2.074(4), O1-C21= 1.287(7), O2-C23 = 1.273(7), C1-Pt1-C5 = 80.0(2), C1-Pt1-O1 = 176.79(19), C1-Pt1-O2 = 93.3(2), C5-Pt1-O2 = 98.53(18), O1-Pt1-O2 = 88.04(15), C1-N1-C2/C4-C5-C7 = 9.3(6).

Single crystals of **3** and **4** were grown via the slow diffusion of cyclohexane into a solution of the complex in dichloromethane. The crystal structures were obtained by X-ray diffraction and confirmed the identity and molecular structures of the targeted compounds (Figure 2a,b).

The solid state structures of **3** and **4** confirm the identities of the target molecules and show that the Pt(acac) moiety is coordinated to the carbene ligand as well as to the C2-position of the pyrene chromophore in **3** and to the C1-position for **4**. Moreover, the X-ray molecular structures of **3** and **4** show that the “acac” ligand chelates the metal center of the cyclometalated (NHC-py)Pt(II) unit (Figure 2). The platinum center in both complexes displays a distorted planar geometry as a result of the coordination to two oxygen atoms (O^O) and to the C^C* chelate of the “Py-NHC” moiety. The dihedral angle between the plane containing the O^O acac ligand and that of the Py-NHC is about 0.6° for **3** and 3.5° for **4**. These data show the near planarity of these cyclometalated platinum complexes [35,40]. Examination of the packing of these complexes in the solid state revealed weak Pt–Pt interactions with contacts at 3.611(1) and 3.696(1) Å for **3** and **4**, respectively [41,42,43,44].

Having fully characterized the novel compounds **3** and **4** we then examined their photophysical properties.

### 2.3. Absorption Properties

**Figure 3:**
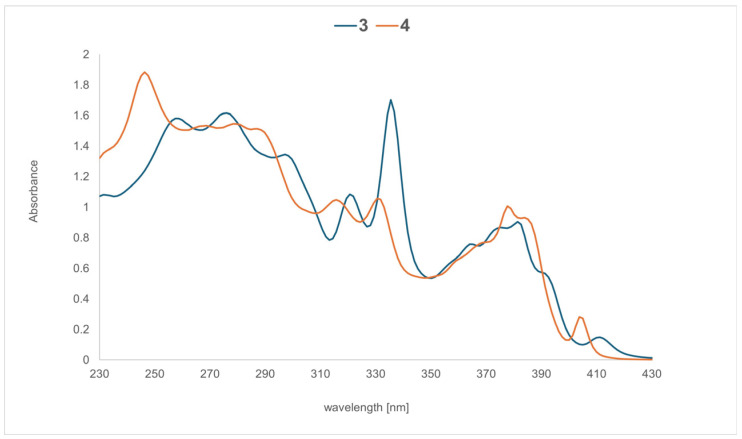
UV/Vis absorbance spectra of complexes **3** and **4**, recorded at room temperature in diluted solutions of dichloromethane (DCM) (c_analyte_ = 5 × 10^−5^ mol L^−1^, d_cuvette_ = 1 cm).

The UV/Vis spectra are depicted in Figure 3. A comparison of the published spectrum of an aminomethyl-substituted pyrene [45] with the measured UV/Vis spectra of compounds **3** and **4** suggests that the absorption bands in the range of 230–350 nm originate from the pyrene chromophore.

The spectrum of **3** is red-shifted in comparison to that of complex **4** by approximately 10 nm. Compound **3** displays its most intense absorption band at 335 nm, accompanied by a shoulder at 320 nm, while the strongest absorption of compound **4** is found at 246 nm followed by an almost plateau-like region, with bands at 265 nm, 280 nm and 290 nm. Starting from the solvent cutoff, the first three absorption bands of complex **3** can be noticed at 257 nm, 276 nm and 300 nm. For complex **4**, characteristic transitions at 315 nm and 330 nm can be observed with different extinction coefficients. Additional significant bands can be found at around 380 nm for both complexes and at 395 nm (**4**) and 411 nm (**3**). The variation in substitution in the 1- or 2-position of the pyrene clearly shows an effect on the absorption spectra and most likely accounts for the observed differences in absorption intensity.

### 2.4. Luminescence Properties

**Figure 4:**
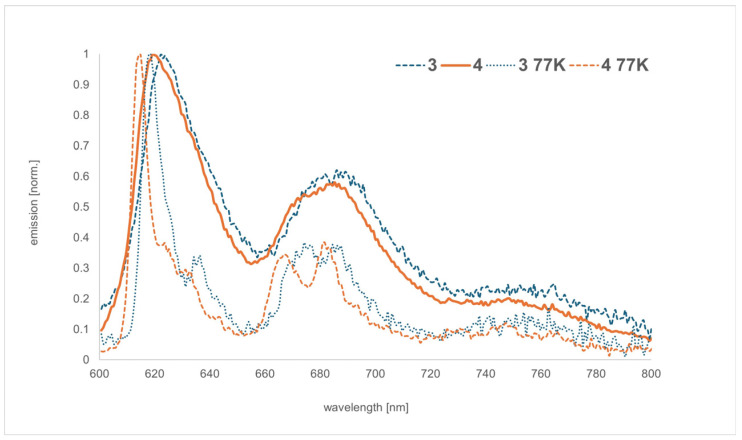
Comparison of normalized room-temperature and 77 K photoluminescence spectra of complexes **3** and **4**, and the photoluminescence emission spectra, recorded under a nitrogen atmosphere at room temperature in poly(methyl methacrylate) (PMMA) films of 60 μm thickness with an emitter concentration of 2 w%. The excitation wavelength (λ_exc_) was 285 nm. Measurements at 77 K were carried out in solutions of 2-methyltetrahydrofuran (2-MeTHF) with an emitter concentration of 5 × 10^−4^ mol L^−1^.

Compounds **3** and **4** also show a weak phosphorescent emission, which was measured at room temperature in PMMA films with a 2 wt% emitter concentration and at 77 K in degassed 2-methyltetrahydrofuran (Figure 4).

The emission spectra of both compounds are very similar under both measurement conditions. In the PMMA matrix, the most intense emission maxima are observed at 622 nm for compound **3** and at 619 nm for compound **4**. These are followed by another emission band at 689 nm and 685 nm, respectively. The corresponding photoluminescence quantum yields at room temperature are only 7% for compound **3** and 17% for compound **4**.

When measured in 2-methyltetrahydrofuran at 77 K, the emission bands become significantly more resolved, indicative of reduced vibronic broadening at low temperature. Under these conditions, more bands can be observed, but due to the weak emission and low quantum yields, a detailed analysis of the orbital contributions does not seem appropriate. The main emission peaks at 618 nm (compound **3**) and 614 nm (compound **4**) are accompanied by shoulders at 636 nm for compound **3** and at 625 nm and 632 nm for compound **4**. In the region between 650 nm and 700 nm, two emission peaks are observed for each compound at low temperature. Compound **3** exhibits peaks at 674 nm and 684 nm, while compound **4** displays corresponding peaks at 666 nm and 681 nm. The quantum yields increase under these conditions to 12% for compound **3** and 26% for compound **4** (Table 1).

This also confirms that the position of the substituent at the pyrene has a significant effect on the quantum yields, but not so much on the emission spectra, as can be seen from Figure 4. The large π–system of the pyrene seems to be responsible for the low quantum yields, similarly to the extension of the π–system in the backbone of the imidazole, as studied earlier [46].

Because of the low quantum yields, we did not measure the decay time at room temperature for complex **3**, but found complex **4** to have a relatively long decay time of 10 μs.

## 3. Conclusions

In this paper we report the preparation of two platinum(II) complexes with the previously known imidazole-pyrenyl ligand, **L1**, and the novel ligand, **L2**, where the imidazole ring is connected to the 2-position of the pyrene chromophore. Upon methylation, related azolium salts **1** and **2** were obtained. The latter were used to prepare two stable cyclometalated *N*-heterocyclic carbene platinum(II) complexes (**3, 4**) with a pyrenyl chromophore and an acetylacetonate ligand. The solid-state structures of the two complexes are described and confirm the identity of the target molecules. To the best of our knowledge these complexes are the first examples of platinum complexes containing a carbene ligand tethered to a pyrene luminophore. At room temperature, both complexes display emissions in the red region of the spectrum. Remarkably, the regioisomer with the Pt-center at the 1-position shows a significantly higher quantum yield compared to the related regioisomer with the metal in the 2-position. These results highlight the importance of the position of the metal at the pyrene rim, which leads to a better overlap between the ligand and the metal orbitals, conferring extra rigidity to the pyrene-system and hence enhancing its emission properties.

## 4. Materials and Methods

Solvents of 99.5% purity were used throughout this study. DMF was dried following standard techniques and kept under an argon atmosphere over molecular sieves (4 Å). Dichloro(1,5-cyclooctadiene)platinum(II) was synthesized following a previously published procedure [47]. 2-Bromopyrene was synthesized from pyrene in two steps in a modified procedure to that used in literature report [39]. 1-(Pyren-1-yl)-1H-imidazole (**L_1_**) and 1-(3-methylimidazolium) pyrene iodide (**1**) were synthesized following the procedure outlined by our group [22,23]. All other chemicals were purchased from usual suppliers and used as received. A Bruker Avance NEO 400 spectrometer was used for recording the ^1^H NMR and ^13^C NMR spectra in the following solvent: DMSO-d^6^, CD_2_Cl_2_, CDCl_3_. Chemical shifts are given in ppm, while coupling constants *J* are given in Hz.

**Synthesis of 1-(pyren-2-yl)-1*H*-imidazole (L_2_).**

To a dried Schlenk tube kept under argon, 2-bromopyrene (1.00 g, 3.55 mmol), imidazole (0.36 g, 5.25 mmol), cesium carbonate (2.30 g, 7.06 mmol), DPM (2,2,6,6-tetramethyl-3,5-heptanedione) (1.31 g, 0.71 mmol) and copper(I) oxide (0.11 g, 0.71 mmol) were introduced. Then, dry DMSO (4 mL) was added through a septum cap under an argon atmosphere. The mixture was stirred for 24 h at 100 °C. Then, the mixture was left to reach room temperature and was poured into a water/ethyl acetate solution (1:3). Subsequent extraction of the aqueous phase with ethyl acetate (5 × 10 mL) followed by a concentration under reduced pressure provided a solid precipitate, which was separated. The product was further purified by recrystallization from diethyl ether/cyclohexane. This creamy compound was dried under vacuum (0.88g, 92%). ^1^H-NMR (400 MHz, Chloroform-d) δ 8.24 (d, J = 15.8 Hz, 3H, H_py_), 8.16 (d, J = 9.0 Hz, 4H, H_py_), 8.09–8.05 (m, 3H), 7.59 (s, 1H, H_im_), 7.37 (s, 1H, H_im_). ^13^C{^1^H}-NMR (101 MHz, Chloroform-*d*^1^) δ 134.9, 132.6, 130.9, 130.2, 129.2, 126.8, 126.5, 126.16, 124.2, 123.7, 117.2. HRMS (ESI) *m*/*z*: [M+H]^+^ Calcd for C_19_H_12_N_2_H 269.1073. Found 269.1072.

**Synthesis of 3-methyl-1-(pyren-1-yl)-1*H*-imidazole-3-ium iodide (2).**

To a flask kept under argon, 1-(pyren-2-yl)-1H-imidazole **L_2_** (0.45 g, 1.68 mmol) and iodomethane (2 mL, 32.1 mmol) were introduced. Then, 5 mL of THF was added and the tube was sealed and heated for 72 h at 70 °C. The reaction evolved to produce a precipitate, which was separated and washed with diethyl ether several times. Then, the compound was dried under vacuum (0.67 g, 97%). ^1^H-NMR (400 MHz, DMSO-d^6^) δ 10.05 (s, 1H, H_im_), 8.70 (s, 2H, H_py_), 8.58 (s, 1H, H_im_), 8.46 (d, J = 8Hz, 2H, H_py_), 8.40 (d, J = 8Hz, 2H, H_py_), 8.30 (d, J = 8Hz, 2H, H_py_), 8.23 (t, J = 8Hz, 1H, H_py_), 8.09 (s, 1H, H_im_), 4.06 (s, 3H, H_Me_). ^13^C NMR (101 MHz, DMSO-d^6^) δ 137.0, 132.8, 132.2, 131.0, 129.9, 127.7, 127.2, 126.8, 125.2, 123.9, 123.5, 122.0, 117.8, 36.8. HRMS(ESI) *m*/*z*: [M]^+^ Calcd for C_20_H_15_N_2_ 283.1230. Found 283.1227.

**Synthesis of complex 3.**

To a dried Schlenk tube kept under argon, 1-(3-methylimidazolium) pyrene iodide (**1)** (0.160 g, 0.4 mmol) and Ag_2_O (0.045 g, 0.2 mmol) were introduced, 10 mL of dry DMF was added, and the reaction mixture was stirred in the absence of light at room temperature for 24 h. Then, [Pt(COD)Cl_2_] (0.15 g, 0.4 mmol) was introduced and the reaction was allowed to proceed for a further 24 h. Later, the mixture was allowed to react at 115 °C for another 24 h. Later, KO*^t^*Bu (0.09 g, 0.8 mmol) and acetylacetone (0.09 mL, 0.8 mmol) were introduced; the reaction mixture was allowed to proceed for 24 h at room temperature and later for 14 h at 100 °C. The solvents were removed under vacuum to generate the residue product. The latter was purified by flash chromatography using diethyl ether/cyclohexane.

The product-containing fraction was dried under vacuum to give a yellow solid (48 mg, 21%). ^1^H-NMR (400 MHz, Methylene Chloride-d^2^) δ 8.65 (s, 1H, H_py_), 8.40 (d, J = 9.4 Hz, 1H, H_py_), 8.16 (d, J = 2.3 Hz, 1H, H_im_), 8.13 (d, J = 7.7 Hz, 1H, H_py_), 8.11–8.03 (m, 3H, H_py_), 7.99–7.90 (m, 2H, H_py_), 7.04 (s, 1H, H_im_), 5.61 (s, 1H, H_acac_), 4.17 (s, 3H, H_Me_), 2.19 (s, 3H, H_Me_), 2.03 (s, 3H, H_Me_). ^13^C{^1^H}-NMR (101 MHz, Methylene Chloride-d^2^) δ 185.5, 185.4, 152.4, 136.5, 131.1, 129.8, 128.6, 127.7, 127.6, 127.6, 126.1, 125.9, 125.6, 125.34, 125.0, 124.2, 123.3, 121.2, 119.4, 118.6, 116.7, 101.9, 87.0, 35.1, 27.8, 27.6. HRMS (ESI) *m*/*z*: [M-H]^+^ Calcd for C_25_H_21_N_2_O_2_Pt 576.1245. Found 576.1246.

**Synthesis of complex 4.**

To a dried Schlenk tube kept under argon, 2-(3-methylimidazolium) pyrene iodide (**2**) (0.160 g, 0.4 mmol) and Ag_2_O (0.045 g, 0.2 mmol) were introduced. Then, 10 mL of dry DMF was added and the reaction mixture was left under stirring at room temperature in the absence of light for 24 h. Later, [Pt(COD)Cl_2_] (0.15 g, 0.4 mmol) and 4 mL 2-butanone were introduced and the reaction was heated to 115 °C for 24 h. After the addition of KO*^t^*Bu (0.09 g, 0.8 mmol) and acetylacetone (0.09 mL, 0.8 mmol), stirring of the mixture was continued for 24 h at room temperature and for 16 h at 100 °C. The solvents were then removed under vacuum to provide a residual product. The latter was purified by column chromatography using diethyl ether/cyclohexane. The compound was dried under reduced pressure to yield an off-white solid (44 mg, 19%). ^1^H NMR (400 MHz, Methylene Chloride-d^2^) δ 9.79 (d, J = 9.5 Hz, 1H, H_py_), 8.11–8.05 (m, 2H, H_py_), 7.99 (d, J = 3.7 Hz, 3H, H_py_), 7.93–7.81 (m, 2H, H_py_), 7.59 (d, J = 2.0 Hz, 1H, H_im_), 6.95 (s, 1H, H_im_), 5.71 (s, 1H, H_acac_), 4.18 (s, 3H, H_Me_), 2.04 (s, 3H, H_Me_), 1.97 (s, 3H, H_Me_). ^13^C{^1^H}-NMR (101 MHz, Methylene Chloride-d^2^) 185.9, 185.2, 132.2, 132.2, 130.3, 129.5, 129.1, 127.9, 127.1, 126.5, 125.6, 125.1, 124.5, 124.4, 124.0, 123.6, 122.9, 115.2, 107.6, 102.7, 85.5, 65.7, 36.2, 28.1, 27.8. HRMS (ESI) *m*/*z*: [M-H]^+^ Calcd for C_25_H_21_N_2_O_2_Pt 576.1245. Found 576.1248.

**X-Ray crystal structure determination.** Crystals suitable for a X-ray diffraction study were chosen, mounted and placed in a cold-nitrogen-gas stream. Intensity data was measured with a Bruker Kappa-APEX2 apparatus equipped with a Cu-Kα radiation fine-focus sealed tube. Bruker APEX software was used for unit-cell parameter determination, data collection strategy implementation, integration and absorption correction. The WinGX framework was used for solving the structure with SHELXT and refining it anisotropically by full-matrix least-squares methods with SHELXL. The structures were sent to the Cambridge Structural Database (deposition numbers CCDC 2483417 for complex **3** and 2483418 for complex **4**) and can be freely retrieved from www.ccdc.cam.ac.uk.

**Crystal data for 3:** C_25_H_20_N_2_O_2_Pt, monoclinic P 2_1_/c, a = 9.6323(6) Å, b = 13.2843(8) Å, c = 15.6033(10) Å, α = γ = 90°, β = 97.999(3)°, V = 1977.1(2) Å^3^, Z = 4, yellow prism 0.15 × 0.05 × 0.03 mm^3^, μ = 13.477 mm^−1^, min/max transmission = 0.35/0.78, T= 200(1) K, λ = 1.54178 Å, θ range = 4.39° to 66.59°, 13762 reflections measured, 3495 independent, R_int_ = 0.0231, completeness = 0.999, 274 parameters, 0 restraints, final R indices R1 [I > 2σ(I)] = 0.0191 and wR2 (all data) = 0.0486, GOF on F^2^ = 1.064, largest difference peak/hole = 0.34 /−0.74 e·Å^−3^.

**Crystal data for 4**: C_25_H_20_N_2_O_2_Pt, monoclinic P 2_1_/c, a = 9.8392(2) Å, b = 15.6315(4) Å, c = 13.4243(3) Å, α = γ = 90°, β = 108.292(1)°, V = 1960.35(8) Å^3^, Z = 4, yellow prism 0.55 × 0.30 × 0.25 mm^3^, μ = 13.592 mm^−1^, min/max transmission = 0.01/0.03, T = 200(1) K, λ = 1.54178 Å, θ range = 4.48° to 66.59°, 13652 reflections measured, 3451 independent, R_int_ = 0.0273, completeness = 0.998, 274 parameters, 0 restraints, final R indices R1 [I > 2σ(I)] = 0.0365 and wR2 (all data) = 0.0974, GOF on F^2^ = 1.175, largest difference peak/hole = 2.16/−1.94 e·Å^−3^.

**Photophysical measurements**. Absorption spectra were performed on a Perkin Elmer Lambda 365 UV/Vis spectrometer in CH_2_Cl_2_ solutions with an analyte concentration of 5 × 10^−5^ mol L^−1^. The phosphorescence decay times were measured on Edinburgh Instruments mini-τ. The excitation was carried out with pulses of an EPLED (360 nm, 20 kHz) and via time-resolved photon counting (TCSPC). The PMMA emitter films were obtained from doctor-blading a solution of the emitter in a 10 wt% PMMA solution in CH_2_Cl_2_ on a quartz substrate with a 60 µm doctor blade. The film was dried and the emission was measured under a N_2_ atmosphere. Measurements at 77 K were conducted in degassed 2-MeTHF (analyte concentration: 5 × 10^−4^ mol L^−1^) in a quartz cuvette. The solution was frozen, and introduced in a quartz finger dewar containing liquid nitrogen. Excitation was conducted in a wavelength range of 250–400 nm (Xe lamp with a monochromator), and the emission and quantum yields were measured with a Quantaurus absolute photoluminescence quantum yield spectrometer from Hamamatsu (model C11347-01) with an integrating sphere.

## Figures and Tables

**Table 1:** Photophysical data of platinum(II) complexes **3** and **4**, measured in a PMMA matrix (2 wt% emitter load, λ_exc_ = 285 nm, RT) and in 2-MeTHF (c = 5 × 10^−4^ mol L^−1^, λ_exc_ = 340 nm, 77 K).

	PL in a PMMA Matrix						PL in 2-MeTHF		
Complex	λ_em_^a^ [nm]	Φ ^b^ [%]	τ_0_^c^ [μs]	k_r_ [×10^3^ s^−1^] ^d^	k_nr_ [×10^3^ s^−1^] ^e^	CIE_xy_^f^	λ_em_^a^ [nm]	Φ ^g^ [%]	CIE_xy_^f^
**3**	622	7	*	*	*	0.489; 0.311	618	12	0.400; 0.261
**4**	619	17	10	105	502	0.528; 0.330	614	26	0.480; 0.259

^a^ Emission peaks. ^b^ PLQY at room temperature and λ_exc_ = 285 nm. ^c^ τ_0_ = τ_exp_ /Φ. ^d^ k_r_ = Φ/τ_exp_. ^e^ k_nr_ = (1 − Φ)/τ_exp_. ^f^ CIE color coordinates. ^g^ PLQY at 77 K and λ_exc_ = 340 nm. * not measured as Φ is below 10%.

## Data Availability

The original contributions presented in this study are included in the article and Supplementary Materials. Further inquiries can be directed to the corresponding authors.

## Supplementary Materials

The following supporting information can be downloaded at https://www.mdpi.com/article/10.3390/molecules30224473/s1: Figures S1–S8: 1H and 13C NMR spectra of all novel compounds, L2, 2, 3 and 4. Absorption spectra of complexes 3 and 4 (Figures S9–S10). Normalized emission spectra of complexes 3 and 4 in PMMA at room temperature and in 2-methyltetrahydrofuran at 77 K (Figures S11–S18).
