# Supplementary material for: Design of a Novel Class of N-Heterocyclic Carbene Cycloplatinated Complexes Containing Pyrene Chromophores
Source: Molecules. 2025 Nov 19;30(22):4473. doi: 10.3390/molecules30224473 (PMC12655006; doi:10.3390/molecules30224473)
Supplement: Supplementary file 1 [file molecules-30-04473-s001.zip › molecules-3962078-supplementary.pdf]

Supporting Information

Design of a Novel Class of *N*-Heterocyclic Carbene Cycloplatinated Complexes Containing Pyrene Chromophores.

Zeping Zhang[1], Yaping Cheng[1], Geoffrey Gontard [1], Tim Riesebeck[2], Sandy Fornal[2], Thomas Strassner[2,*], and Hani Amouri [1,*]

1 Sorbonne Université- Campus Pierre et Marie Curie, Institut Parisien de Chimie Moléculaire (IPCM) UMR CNRS 8232, 4 place Jussieu, 75252 Paris cedex 05, France; zeping.zhang@sorbonne-universite.fr; yaping.cheng@sorbonne-universite.fr; geoffrey.gontard@sorbonne-universite.fr; hani.amouri@sorbonne-universite.fr.

2 Physikalische Organische Chemie, Technische Universität Dresden, 01069 Dresden, Germany; sandy.fornal@tu-dresden.de; tim_niklas.riesebeck@tu-dresden.de; thomas.strassner@tu-dresden.de.

* Correspondence: TS: thomas.strassner@tu-dresden.de;
HA: hani.amouri@sorbonne-universite.fr

TABLE OF CONTENTS

Figures S1-S8 (^{1}H, ^{13}C) -NMR spectra of compounds (**L2**, **2**, **3** and **4**).

Figures S9-S10 UV/Vis spectra of complexes **3** and **4**.

Figures S11-S18 Normalized emissions of **3** and **4** in PMMA at room temperature and in 2-methyltetrahydrofuran at 77K.

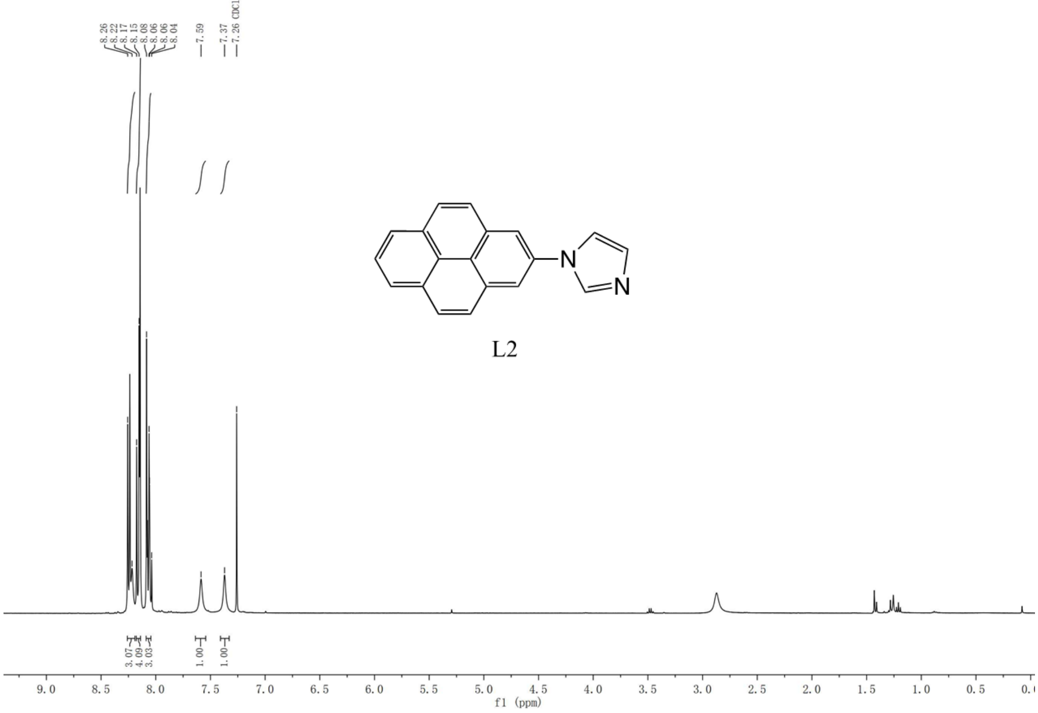

Figure S1. ^{1}H NMR of compound **L2** in $CDCl_3$

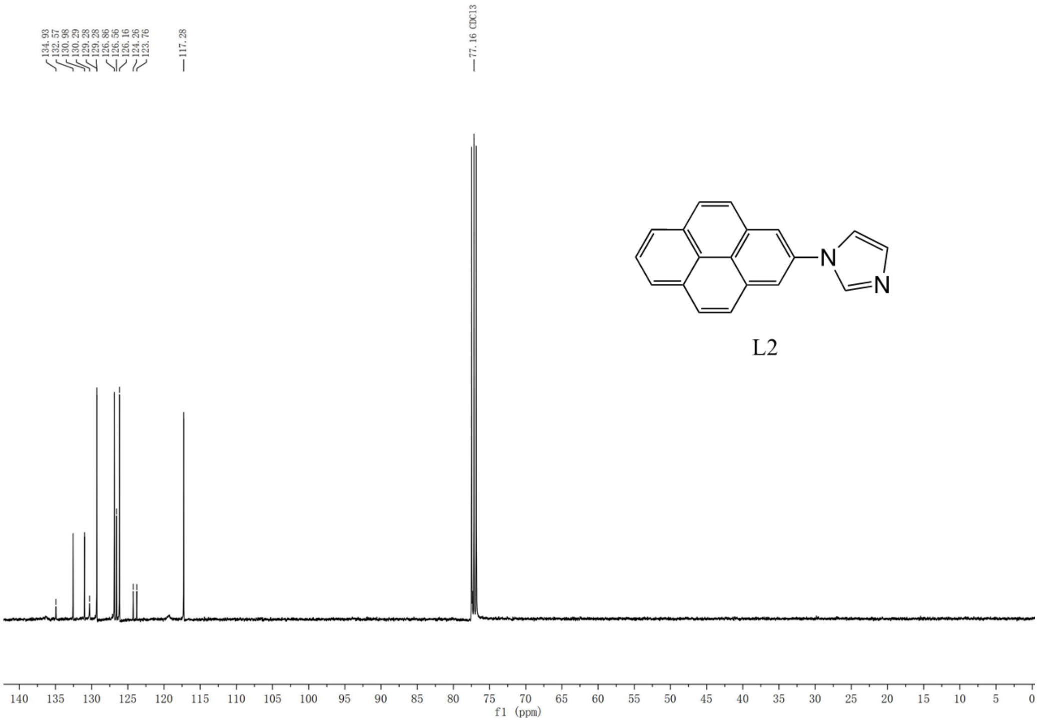

Figure S2. ^{13}C NMR of compound **L2** in $CDCl_3$

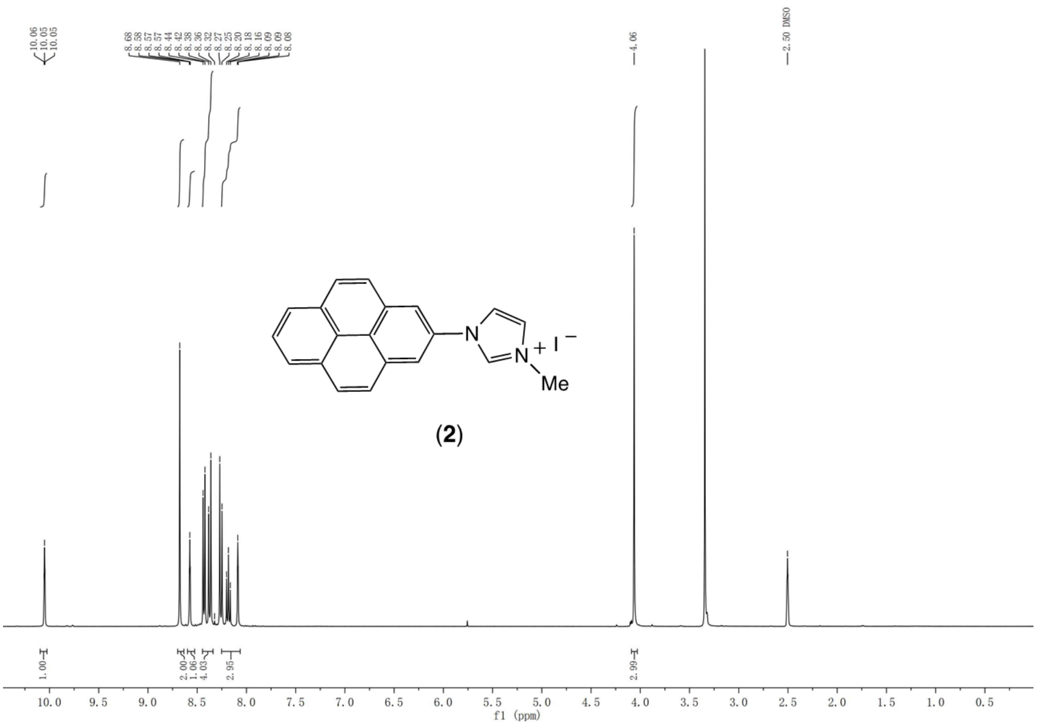

Figure S3. ^{1}H NMR of compound **2** in DMSO

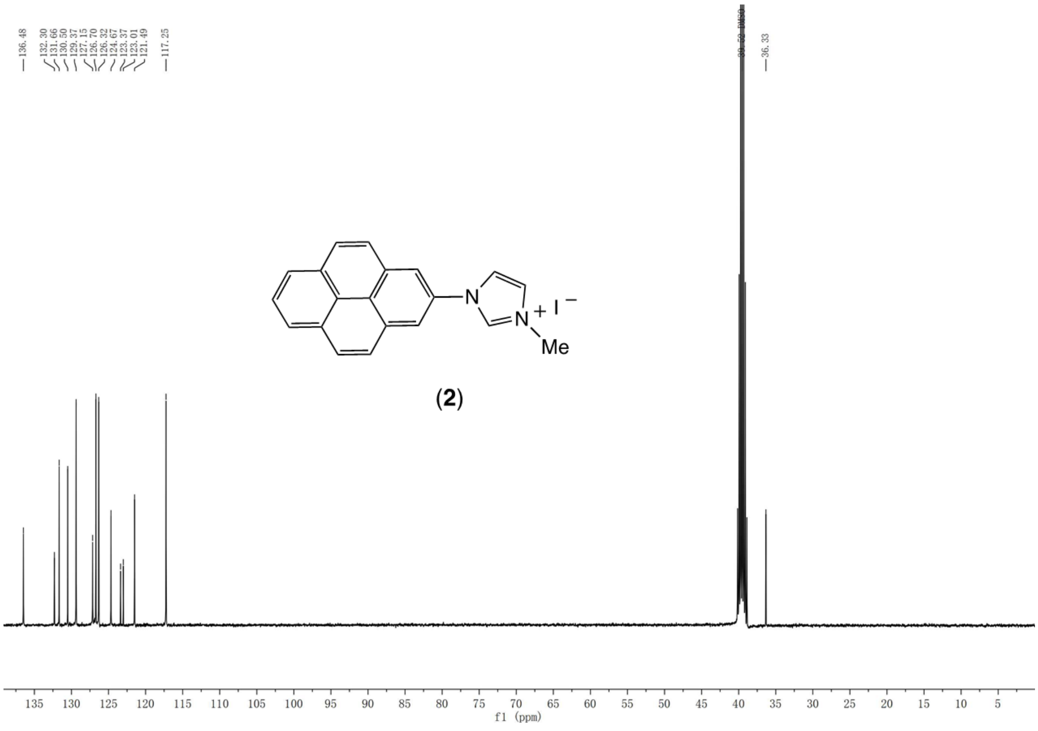

Figure S4. ^{13}C NMR of compound **2** in DMSO

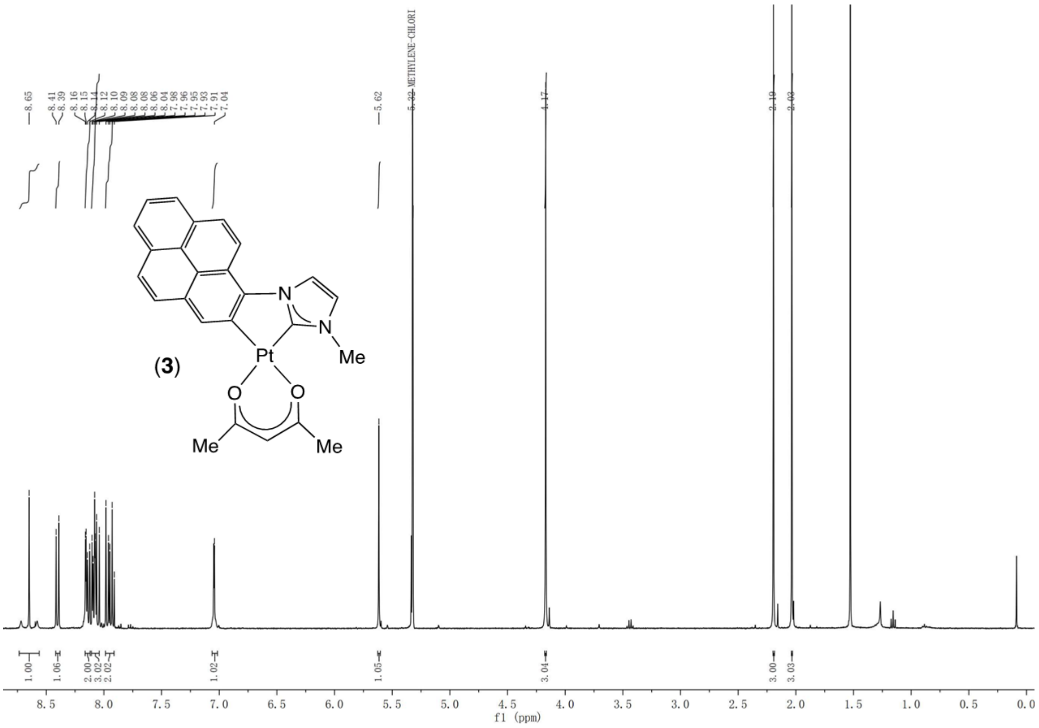

Figure S5. ^{1}H NMR of compound **3** in CD_2Cl_2

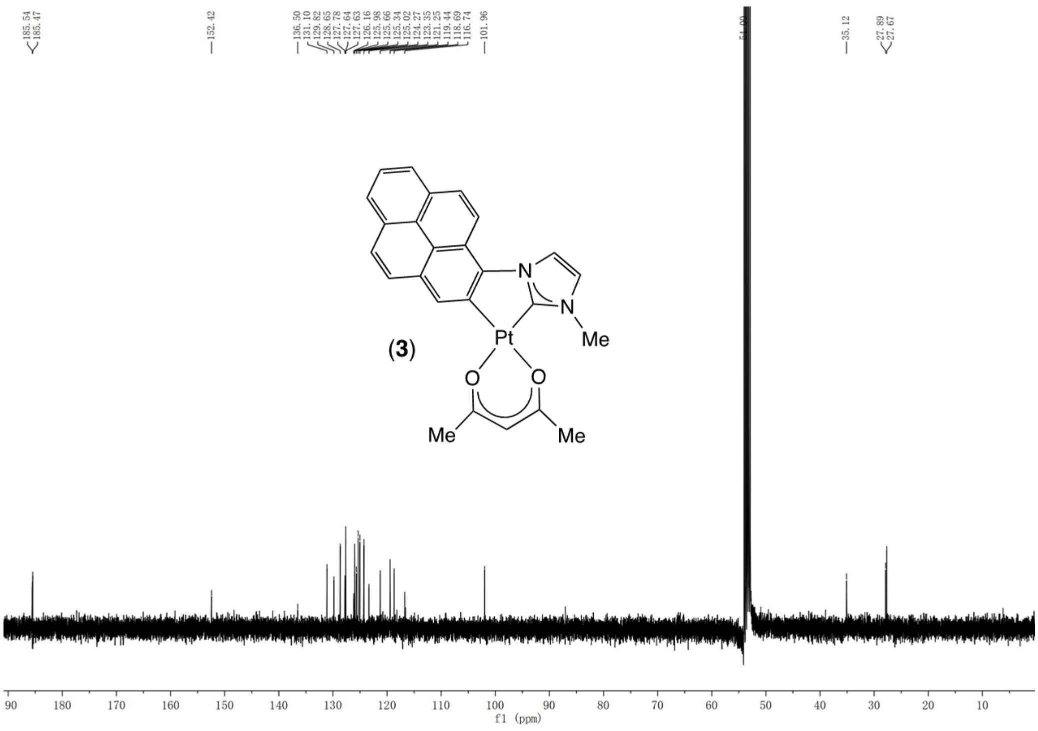

Figure S6. ^{13}C NMR of compound **3** in CD_2Cl_2

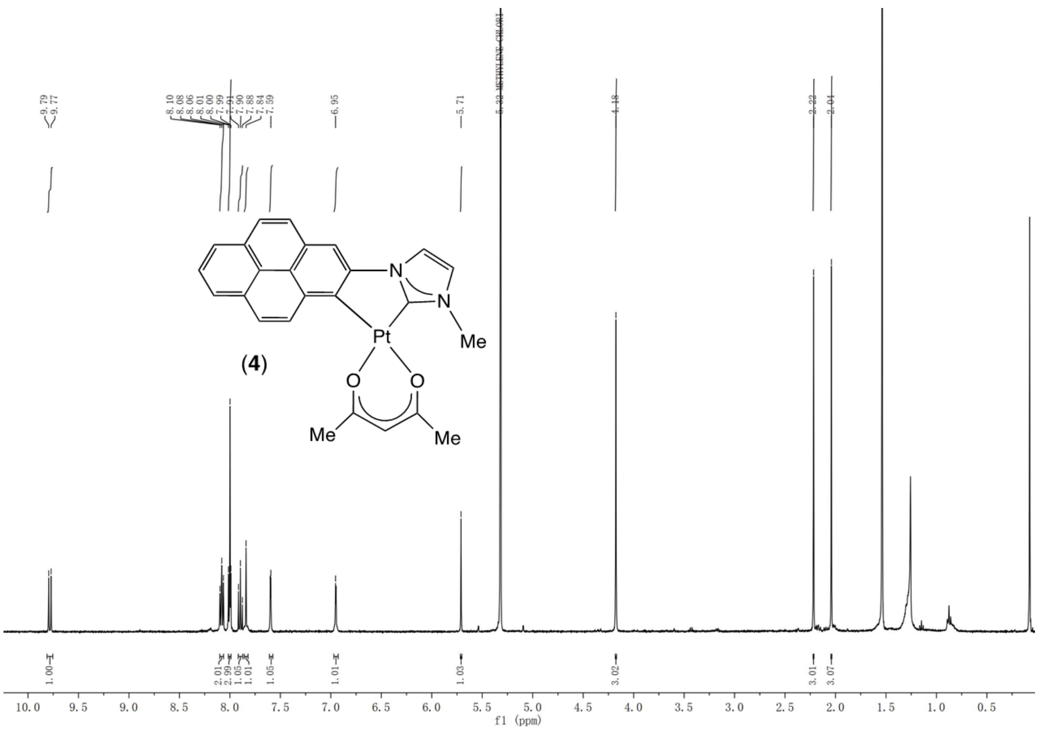

Figure S7. ^{1}H NMR of complex **4** in CD_2Cl_2

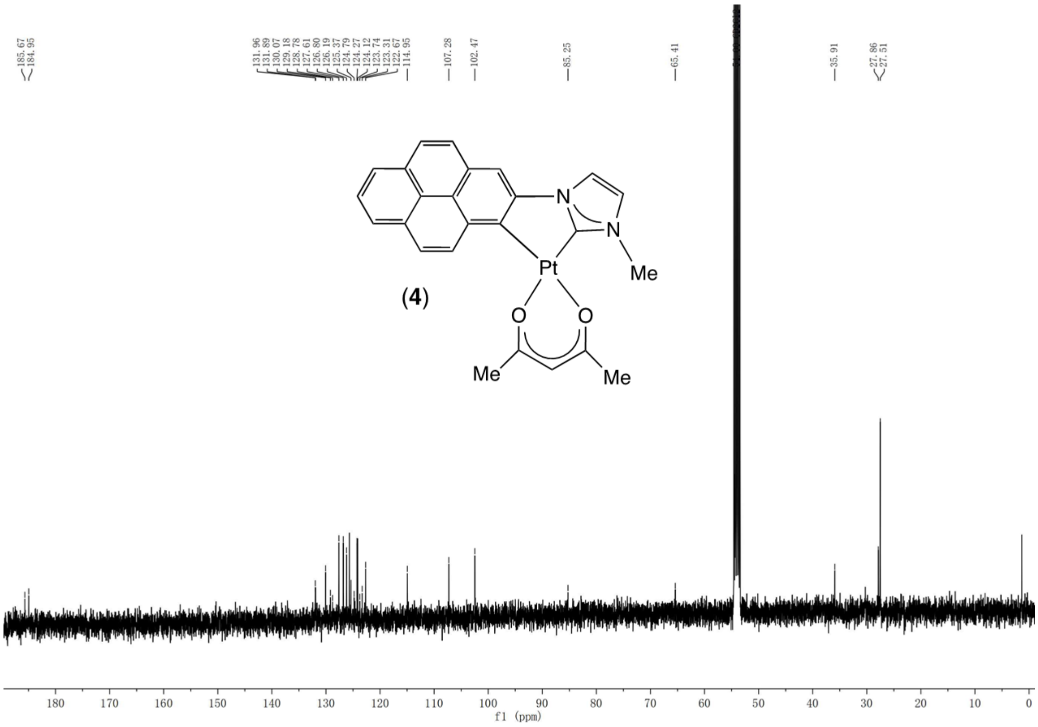

Figure S8. ^{13}C NMR of complex **4** in CD_2Cl_2

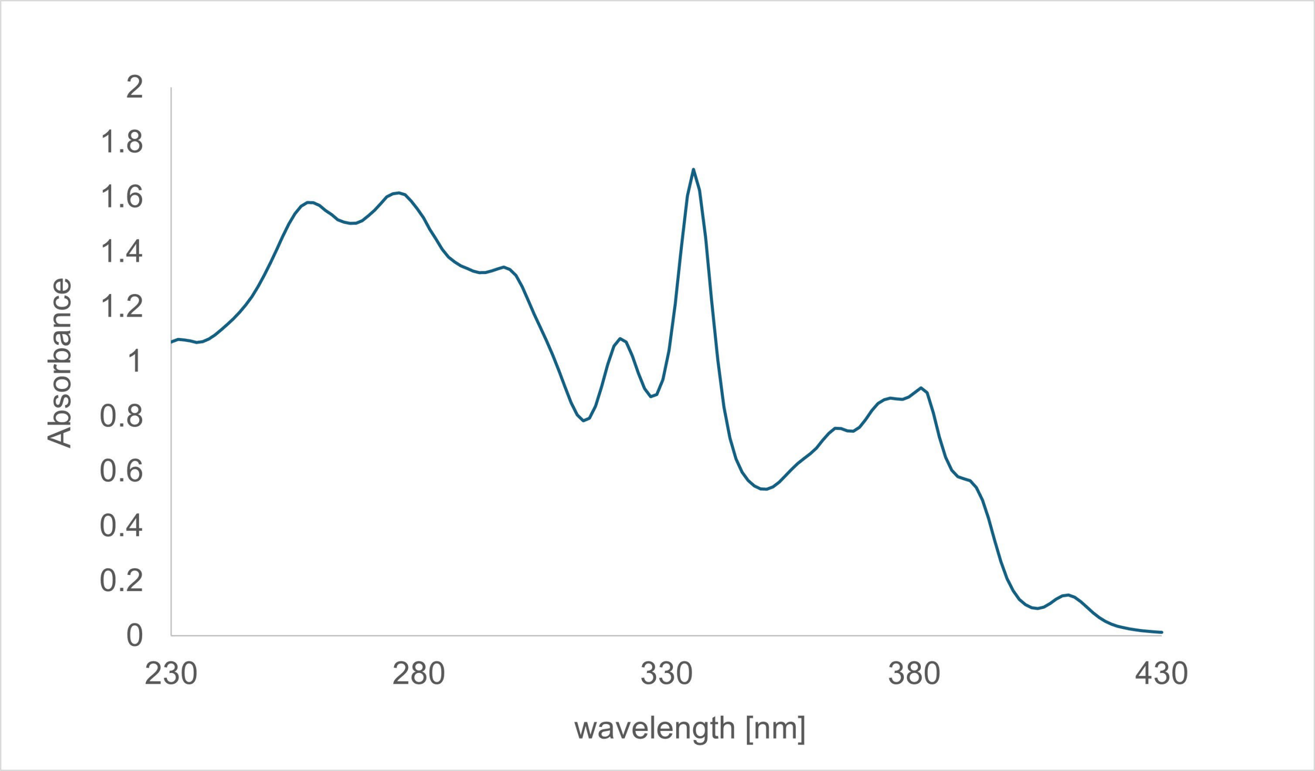

Figure S9. UV/Vis spectrum of complex **3** in CD_2Cl_2 (c=5x10^{-5} mol L^{-1}) at room temperature

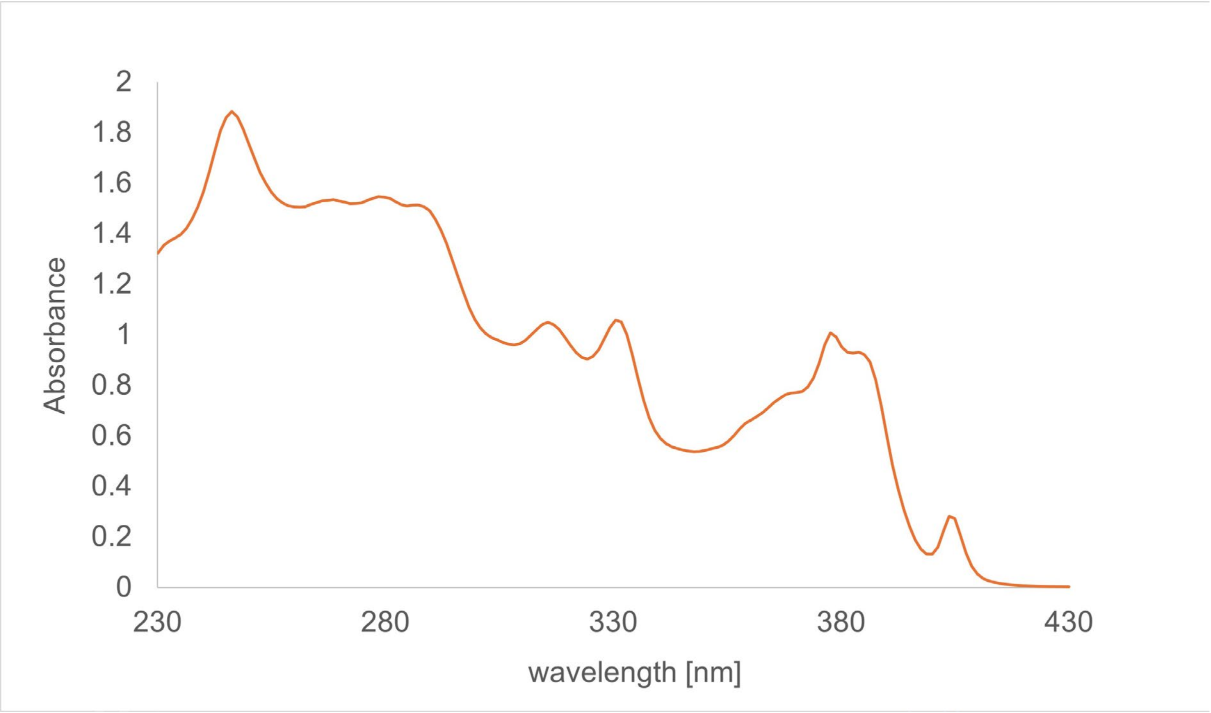

Figure S10. UV/Vis spectrum of complex **4** in CD_2Cl_2 (c=5x10^{-5} mol L^{-1}) at room temperature

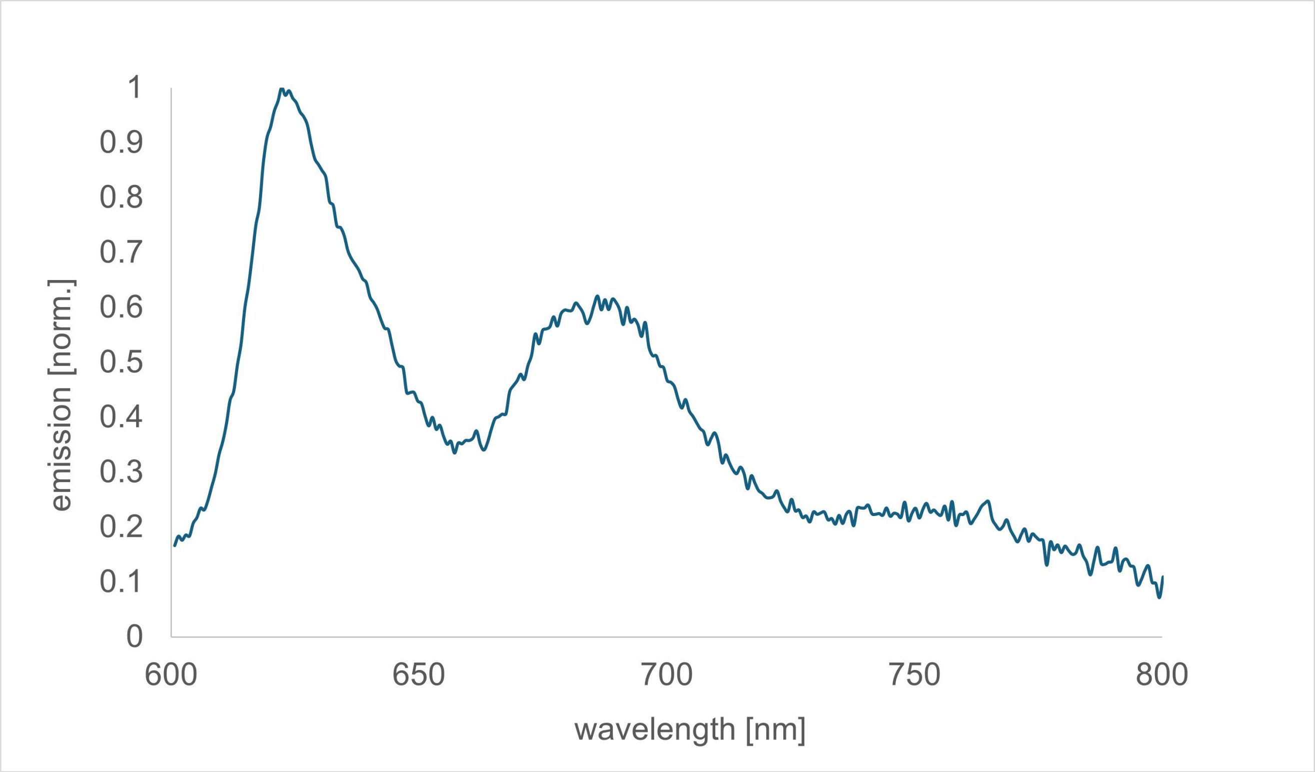

Figure S11. Normalized emission spectrum of complex **3** in PMMA film (emitter concentration of 2 w%; 60 μm thickness; λ_{exc} = 285 nm) at room temperature

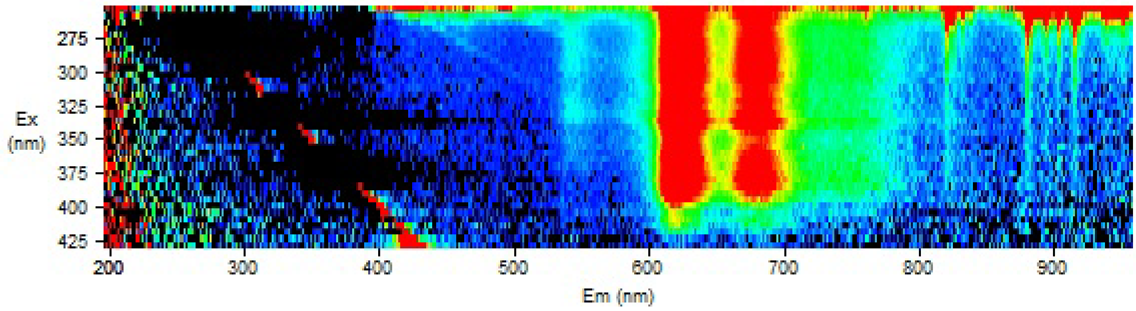

Figure S12. 2D Plot of emission spectra of complex **3** in PMMA film (emitter concentration of 2 w%; 60 μm thickness) at room temperature

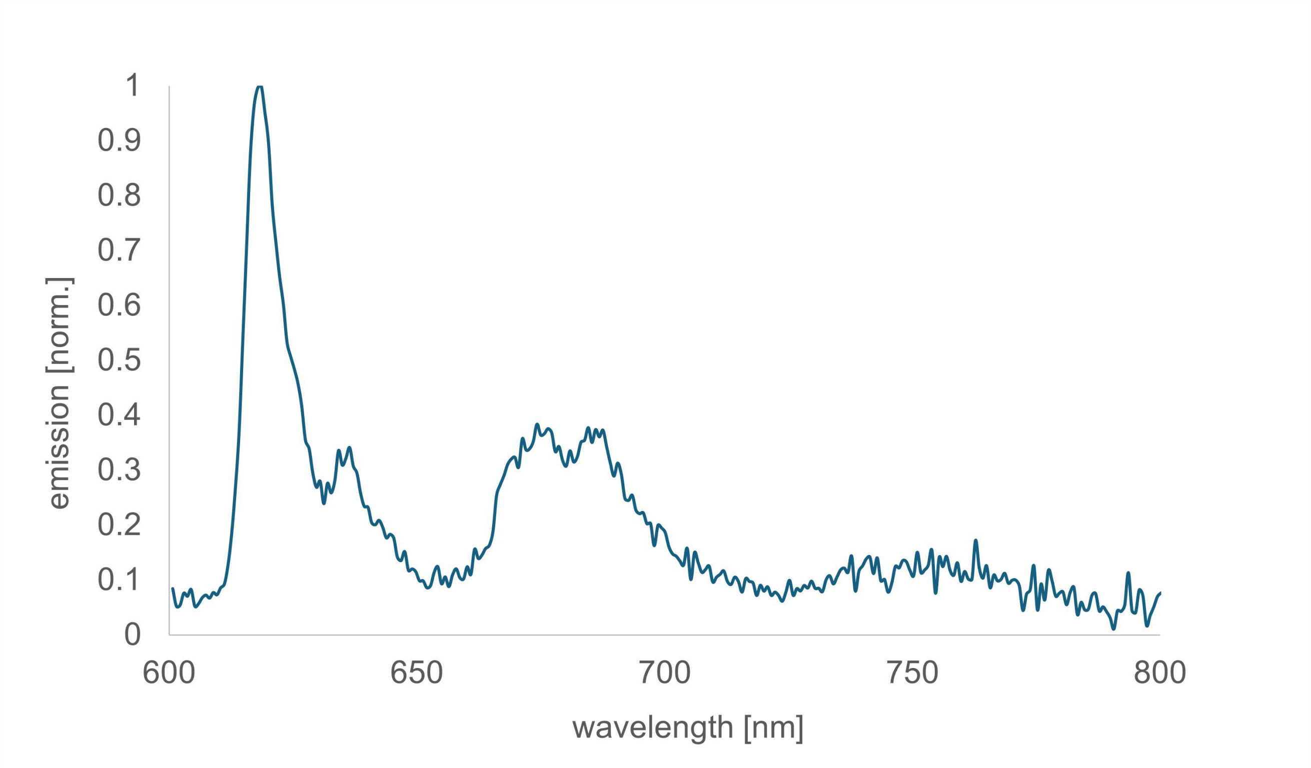

Figure S13. Normalized emission spectrum of complex **3** in 2-methyltetrahydrofuran ($c=5x10^{-4}$ mol L^{-1}; λ_{exc} = 340 nm) at 77K

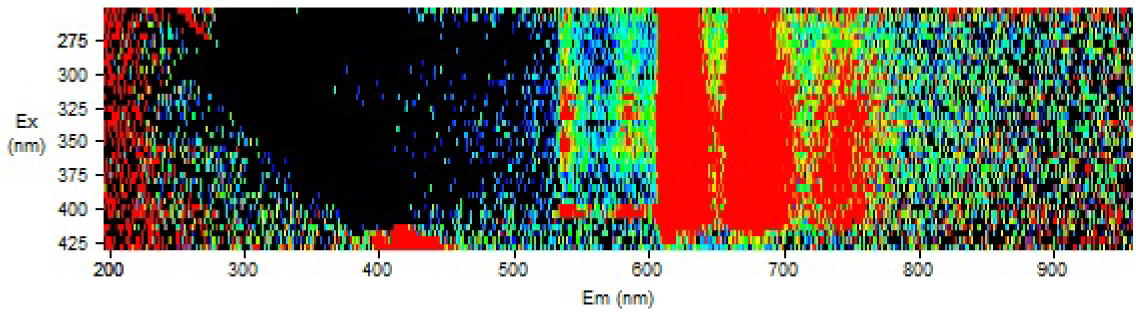

Figure S14. 2D Plot of emission spectra of complex **3** in 2-methyltetrahydrofuran ($c=5x10^{-4}$ mol L^{-1}) at 77K

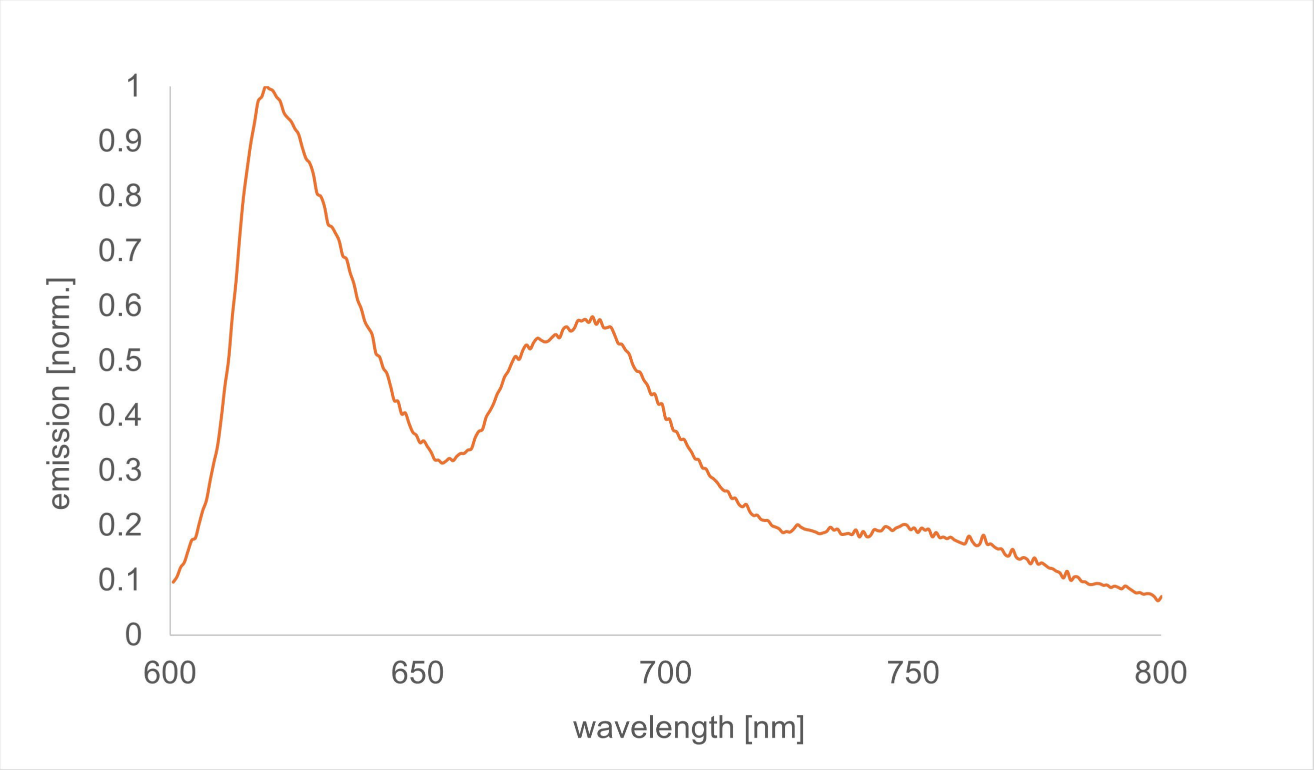

Figure S15. Normalized emission spectrum of complex **4** in PMMA film (emitter concentration of 2 w%; 60 μM thickness) at room temperature

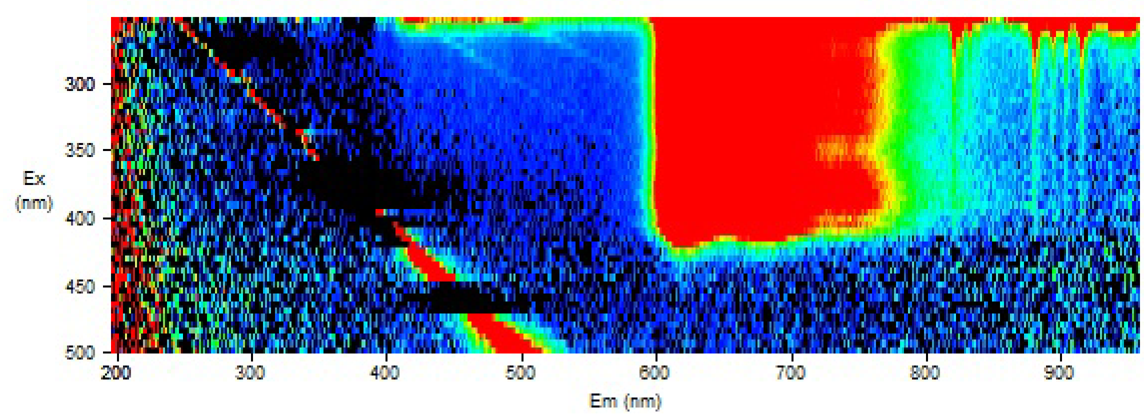

Figure S16. 2D Plot of emission spectra of complex **4** in PMMA film (emitter concentration of 2 w%; 60 μm thickness) at room temperature

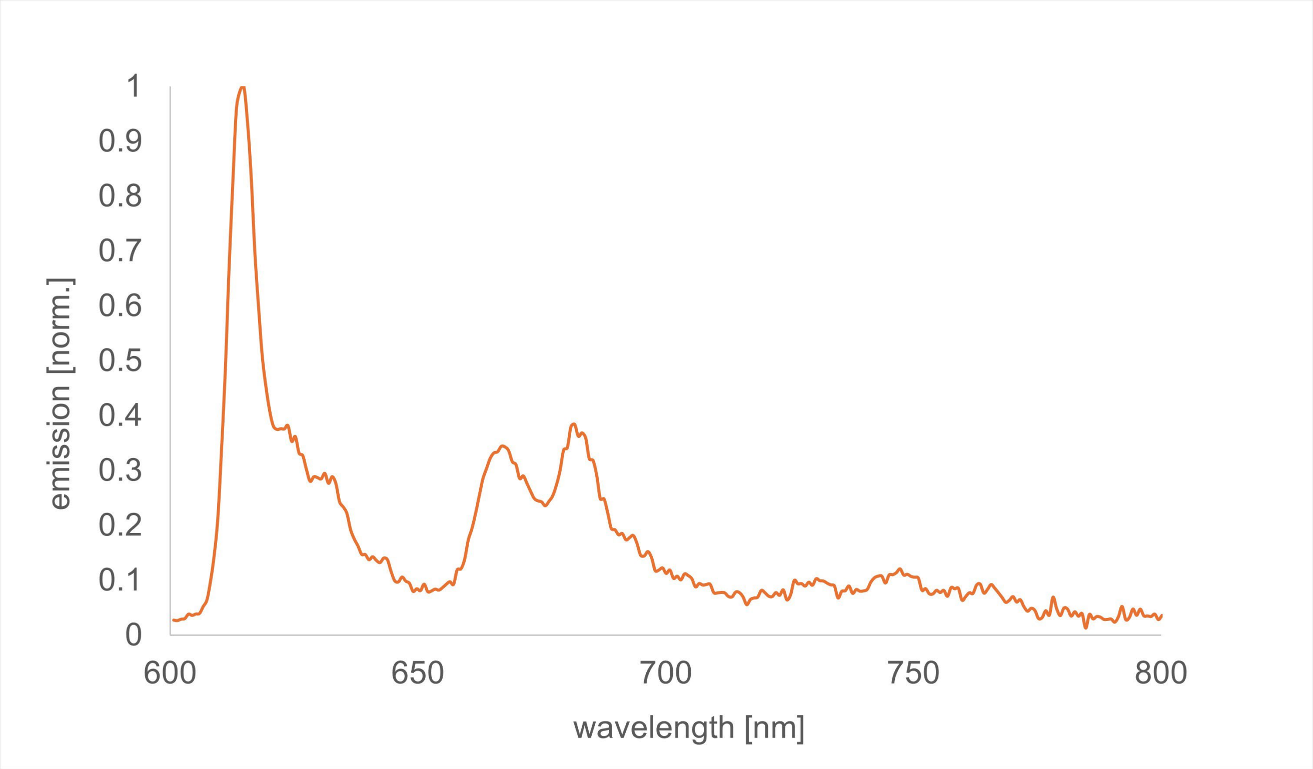

Figure S17. Normalized emission spectrum of complex **4** in 2-methyltetrahydrofuran ($c=5x10^{-4}$ mol L^{-1}; λ_{exc} = 340 nm) at 77K

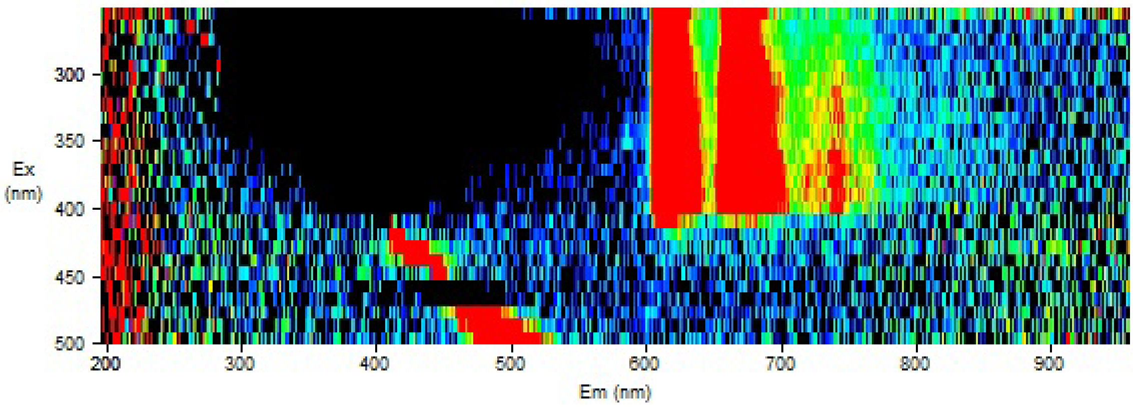

Figure S18. 2D Plot of emission spectra of complex **4** in 2-methyltetrahydrofuran ($c=5x10^{-4}$ mol L^{-1}) at 77K
